# Linoleic acid metabolic reprogramming is linked to immunometabolic remodeling and post-transplant recurrence risk in hepatocellular carcinoma

**DOI:** 10.3389/fimmu.2026.1862165

**Published:** 2026-08-11

**Authors:** Ruixin Zhang, Anhong Zhang, Tian Gao, Chengjie Zhang, Yiqian Liu, Lixin Liu

**Affiliations:** 1 The First Clinical Medical College, Shanxi Medical University, Taiyuan, China; 2 Department of Hepatobiliary Surgery & Liver Transplantation Center, The First Hospital of Shanxi Medical University, Taiyuan, China; 3 Translational Medicine Research Center, Shanxi Medical University, Taiyuan, China; 4 Department of Gastroenterology and Hepatology, The First Hospital of Shanxi Medical University, Taiyuan, China; 5 Key Laboratory of Prevention and Treatment of Liver Injury and Digestive System Neoplasms, Provincial Committee of the Medical and Health, Taiyuan, China

**Keywords:** fatty acid desaturation, fatty acid oxidation, hepatocellular carcinoma, immunometabolism, linoleic acid, liver transplantation, neoplasm recurrence, regulatory T cells

## Abstract

**Background:**

Lipids serve as both metabolic substrates and signaling mediators that critically regulate immune cell fate, function, tolerance, and intercellular communication. In hepatocellular carcinoma (HCC), it remains unclear how intratumoral linoleic acid (LA) allocation between desaturation and oxidative catabolism influences immunometabolic remodeling and post-transplant recurrence.

**Methods:**

Multi-omics profiling was conducted in a liver transplant-associated HCC cohort using paired tumor/non-tumor tissues and serum samples. The intratumoral lipid milieu (C4–C24 fatty acids) was quantified by targeted gas chromatography, whereas lipid–immune programs were resolved by single-nucleus RNA sequencing (snRNA-seq) integrated with targeted transcriptomic profiling. Key immune phenotypes and candidate genes were validated by serum inflammatory mediator profiling, flow cytometric lymphocyte immunophenotyping, spatially resolved tissue assays, and qRT–PCR. LA perturbation experiments provided functional support *in vitro*.

**Result:**

Fatty acid profiling revealed that non-recurrent tumors were characterized by a higher intratumoral LA/AA ratio and a lower estimated Δ6-desaturation index than recurrent tumors, whereas recurrent tumors exhibited a higher estimated Δ6-desaturation index than their matched distant non-tumor liver tissues. snRNA-seq identified fatty acid catabolism as the most prominently downregulated metabolic pathway in recurrent tumors; malignant-cell analysis further highlighted EHHADH and ACSL3 as key genes involved in recurrence-associated lipid metabolic remodeling. *In vitro*, exogenous LA-BSA increased ROS accumulation and reduced SPP1 and NECTIN2 expression in HCC cells. Cell–cell communication analysis showed enhanced tumor–Treg signaling in recurrence, with VISTA- and CD80-related networks shifting from Kupffer cells toward SPP1^+^ macrophages and Tregs. Peripheral and tissue immunophenotyping showed reduced CD8^+^ T cells and higher IL-10 in recurrent tumors.

**Conclusion:**

Multi-omics analyses indicate that, in primary pre-transplant HCC, post-transplant recurrence is associated with an imbalance between a higher estimated Δ6-desaturation index and attenuated oxidative catabolism-related features of linoleic acid, accompanied by Treg-associated immunosuppressive remodeling. These findings provide a biologically informed framework for post-transplant recurrence risk stratification and further translational investigation.

## 1 Introduction

Hepatocellular carcinoma (HCC), which accounts for 85%–90% of primary liver cancers, ranks as the third leading cause of cancer-related mortality globally (1, 2). Transplantation has evolved into a well-established curative treatment for patients with hepatocellular carcinoma who meet established selection criteria (3). Nevertheless, post-transplant tumor recurrence remains a key determinant of long-term survival, with a median post-recurrence survival of 17 months (4). Increasing evidence indicates that, under conditions of long-term postoperative immunosuppression, the risk of tumor recurrence following liver transplantation is predominantly governed by the intrinsic biological characteristics of the primary tumor prior to transplantation (5). However, the immunometabolic features of the primary tumor that are associated with post-transplant recurrence remain poorly defined.

Lipid metabolic reprogramming is increasingly recognized as a critical contributor to tumor progression, involving coordinated changes in cellular energy metabolism, membrane composition, redox homeostasis, and lipid-derived signaling pathways (6). Linoleic acid (LA; 18:2n-6) is a major dietary ω-6 essential polyunsaturated fatty acid that serves not only as a structural lipid component in tumors but also as a precursor for bioactive lipid mediators regulating inflammation and immunity (7, 8). Notably, the metabolic partitioning of LA across competing pathways, rather than its absolute tissue abundance, may play a more important role in disease progression (9). ACSL3 (acyl-CoA synthetase long-chain family member 3) activates long-chain fatty acids by converting them to acyl-CoA esters, enabling their use in *de novo* lipogenesis, phospholipid remodeling, protein lipoylation, and mitochondrial β-oxidation (10). Previous studies show that, after activation and incorporation into the intracellular acyl-CoA pool, LA enters arachidonic acid (AA) metabolism through delta-6 desaturation to generate immunomodulatory lipid mediators, including prostaglandins, that shape the tumor immune microenvironment (11). In contrast, EHHADH (enoyl-CoA hydratase and 3-hydroxyacyl-CoA dehydrogenase) encodes a peroxisomal bifunctional enzyme that catalyzes key steps in long-chain fatty-acid β-oxidation, thereby contributing to fatty acid catabolism and modulating oxidative substrate handling and lipid-derived signaling (12, 13). Distinct metabolic pathways exert divergent effects on lipid signaling and tumor-associated immune remodeling. However, it remains unclear how intratumoral LA is partitioned between FADS2-associated desaturation and EHHADH-associated oxidative disposal, and whether this balance is linked to recurrence risk in HCC.

Liver transplantation necessitates lifelong immunosuppression to prevent allograft rejection, thereby compromising antitumor immune surveillance (14). In recipients transplanted for HCC, this immunosuppression may promote tumor immune escape and is strongly implicated in early post-transplant recurrence, in which immune reprogramming plays a central role (15). Regulatory T cells (Tregs) are central to peripheral immune tolerance and maintain immune homeostasis in hepatocellular carcinoma and liver transplantation (16). There, they restrain effector T-cell responses, suppress local inflammation, and tip the balance between anti-tumor immunity and transplant tolerance (17). Recent studies suggest that metabolic reprogramming can shape the expansion, recruitment, and immunosuppressive phenotype of Tregs (18). In HCC, PON1 upregulation promotes HIF-1α degradation, inhibits glycolysis and lactate production, and is associated with Treg enrichment (19). Furthermore, knockout of the rate-limiting enzyme in sphingolipid synthesis, Sptlc2, reduces intratumoral Treg accumulation and inhibits tumor growth (20). However, whether metabolic reprogramming affects HCC recurrence risk by regulating Tregs in liver transplant recipients with long-term immunosuppression remains unclear.

In summary, it remains unclear whether differential LA allocation in primary HCC is associated with tumor immune remodeling, and whether such immunometabolic features are linked to the risk of early post-transplant recurrence in the setting of iatrogenic immunosuppression. Given the scarcity of single-cell studies specifically addressing post-transplant HCC recurrence, an integrated clinical and multi-omic approach may help identify biologically relevant recurrence-associated immunometabolic features. Therefore, in a 5-year follow-up cohort of HCC liver transplant recipients, we integrated single-cell profiling and quantitative fatty acid analysis to characterize recurrence-associated metabolic–immune remodeling, thereby providing a framework for immunometabolic risk stratification and translational investigation in the post-transplant setting.

## 2 Materials and methods

### 2.1 Clinical samples and cohort design

This study collected clinical data and liver transplant specimens from patients with HCC who underwent liver transplantation at the First Hospital of Shanxi Medical University between April 2020 and April 2025. It was a single-center cohort study with prospective specimen collection and longitudinal follow-up. According to predefined inclusion and exclusion criteria, a total of 5 patients with postoperative recurrence and 23 patients without recurrence were enrolled. The patient selection process is illustrated in **Supplementary Figure 1**. Tissue samples were prospectively collected from two distinct anatomical regions: tumor tissue and distant normal liver tissue (≥5 cm from the tumor margin). All patients underwent follow-up, during which recurrence status was determined based on imaging findings and serum alpha-fetoprotein (AFP) levels, leading to classification into recurrent and non-recurrent groups. Baseline clinical characteristics are summarized in **Supplementary Table 1**.

The primary endpoint of this study was recurrence-free survival. Specifically, the observation period was defined as the time from surgery to first recurrence for patients who experienced relapse and as the time from surgery to last follow-up for those without recurrence. All patients completed scheduled postoperative follow-up. Among those without recurrence, median follow-up was 47.5 months (IQR: 32.4-54.4), with all exceeding 24 months.

### 2.2 Fatty acid measurement

Tissue lipids were hydrolyzed, extracted, methylated to FAMEs, and analyzed on an Agilent 7890A GC with a 100 m × 0.25 mm × 0.2 µm cyanopropyl capillary column; nitrogen carrier; injector 250 °C; detector 260 °C. Triundecanoin served as the internal standard. A 37-species fatty acid panel was quantified; reagents are listed in **Supplementary Table 2**. Product-to-precursor ratios served as composition-based proxy indices for individual pathway steps.

### 2.3 Calculation of fatty acid-derived indices

Fatty acid-derived indices were calculated from tissue fatty acid composition. The estimated Δ6-desaturation index was defined as γ-linolenic acid/linoleic acid (GLA/LA; C18:3n-6/C18:2n-6), the elongation index as dihomo-γ-linolenic acid/γ-linolenic acid (DGLA/GLA; C20:3n-6/C18:3n-6), and the estimated Δ5-desaturation index as arachidonic acid/dihomo-γ-linolenic acid (AA/DGLA; C20:4n-6/C20:3n-6). AA/(LA + GLA + DGLA) and AA/total n-6 PUFA ratios were further calculated to assess the relative distribution of AA within the n-6 PUFA pathway and the total n-6 PUFA pool. These indices were used as surrogate composition-based measures.

### 2.4 Single-nucleus RNA sequencing

Snap-frozen tissues were lysed in sucrose buffer with RNase inhibition and purified by density gradient centrifugation to isolate nuclei. snRNA-seq libraries were prepared using the 10x Genomics Chromium Single Cell 3′ v3 kit and sequenced on an Illumina NovaSeq 6000. Reads were processed with Cell Ranger (v3.1.0; GRCh38/hg38) to generate feature–barcode matrices. Downstream analysis was performed in Seurat, including quality control, normalization, dimensionality reduction, clustering, and UMAP visualization.

### 2.5 Copy number variation inference

CNV inference was performed for hepatocyte-lineage cells using the inferCNV algorithm based on single-nucleus transcriptomic profiles. Non-epithelial cells, including immune and stromal cells, were used as reference populations to establish the baseline expression profile. Gene expression values were ordered according to their chromosomal positions to infer large-scale chromosomal copy-number alterations. Based on the relative inferCNV-derived CNV burden, hepatocyte-lineage cells were classified into high-CNV malignant cells and low-CNV malignant cells.

### 2.6 Immunohistochemistry and immunofluorescence

IHC and IF were performed on tumor, adjacent non-tumor, and distant normal live tissues as specified for each marker. After standard processing, sections were incubated with primary antibodies against EHHADH, ACSL3, and CD8 (IHC) or CD4, FOXP3, and TNF-α (IF) overnight at 4 °C. For IHC, HRP-conjugated secondary antibodies with DAB detection and hematoxylin counterstaining were used. For IF, fluorophore-conjugated secondary antibodies and DAPI nuclear staining were applied. Staining was quantified as integrated density (IntDen). Antibody details are in **Supplementary Table 3**.

### 2.7 Quantitative real-time PCR

Total RNA was extracted from tissue samples using TRIzol reagent, and its concentration and purity were assessed. The reaction mixture included cDNA template, gene-specific primers (**Supplementary Table 4**), SYBR Green Master Mix, and nuclease-free water. Cycling conditions were 95 °C for 5 minutes, followed by 45 cycles of 95 °C for 15 seconds and 60 °C for 30 seconds. Melting curve analysis verified amplification specificity. Each sample was run in triplicate, and gene expression was analyzed using the 2^−ΔΔCt^ method.

### 2.8 Flow cytometry-based immune assessment

Circulating lymphocyte subset data and postoperative serum cytokine data were extracted from routine clinical laboratory records. Peripheral blood lymphocyte subsets were assessed by flow cytometry before and after transplantation, whereas serum cytokines were measured postoperatively using a flow cytometry-based cytokine assay. All measurements were performed by the institutional clinical laboratory according to standardized clinical protocols.

### 2.9 Public datasets and external validation

Gene expression matrices and clinical data were downloaded from GSE249913 (21). Differential expression analysis was performed using GEO2R (limma package). For TCGA-LIHC, STAR-generated RNA-seq counts and clinical information were retrieved from the GDC Data Portal (https://portal.gdc.cancer.gov). Counts were converted to TPM and log_2_-transformed as log2 (TPM + 1), yielding 367 cases for downstream analysis.

### 2.10 Cell lines and culture conditions

Human hepatocellular carcinoma cell lines Huh7 and HCCLM3 were obtained from Zhejiang Nuobo Biological Products Co., Ltd. and cultured in high-glucose Dulbecco’s Modified Eagle Medium (DMEM) supplemented with 10% (v/v) fetal bovine serum (FBS) and 1% (v/v) penicillin–streptomycin solution at 37 °C in a humidified atmosphere containing 5% CO_2_. Cells were routinely tested and confirmed to be mycoplasma-free.

### 2.11 Linoleic acid treatment

Linoleic acid (LA; Aladdin) was conjugated to fatty acid-free BSA (FAF-BSA) to form an LA-BSA complex. Huh7 and HCCLM3 cells were treated with 75-125 μM LA (in FAF-BSA) for 72 h; vehicle controls received FAF-BSA alone at equivalent volumes.

### 2.12 CCK-8 assay

Cell viability was assessed using the Cell Counting Kit-8 (CCK-8; Sevenbio). Huh7 and HCCLM3 cells (2-3×10^3^ per well) were seeded in 96-well plates. After LA or vehicle treatment for 24, 48, or 72 h, 10 μL of CCK-8 reagent was added to each well and incubated at 37 °C. Absorbance was measured at 450 nm.

### 2.13 Reactive oxygen species measurement

Intracellular ROS were measured in Huh7 and HCCLM3 cells using DCFH-DA (Beijing Solarbio Science & Technology Co., Ltd.). Cells were seeded in 24-well plates, treated with linoleic acid or vehicle for 72 h. After treatment, cells were washed with PBS, incubated with DCFH-DA in serum-free medium at 37 °C for 20 min in the dark, washed again to remove unincorporated probe, and imaged by fluorescence microscopy.

### 2.14 Statistical analysis

Statistical analyses were performed using R (v4.0.3) and GraphPad Prism (v9). To address small-sample uncertainty, robust nonparametric methods were preferentially applied to continuous variables, and selected biologically informed within-sample ratios were further analyzed to reduce inter-individual variability. Comparisons between two independent groups were conducted using the Mann–Whitney U test, and comparisons among three or more groups were performed using the Kruskal–Wallis test. Categorical variables were analyzed using Fisher’s exact test. Correlations were assessed using Spearman’s rank correlation coefficient. Survival was evaluated using the Kaplan–Meier method and compared using the log-rank test. Statistical significance was defined as *P* < 0.05.

## 3 Results

### 3.1 Linoleic acid–related remodeling of n-6 polyunsaturated fatty acids is associated with post-transplant recurrence

**Figure 1:**
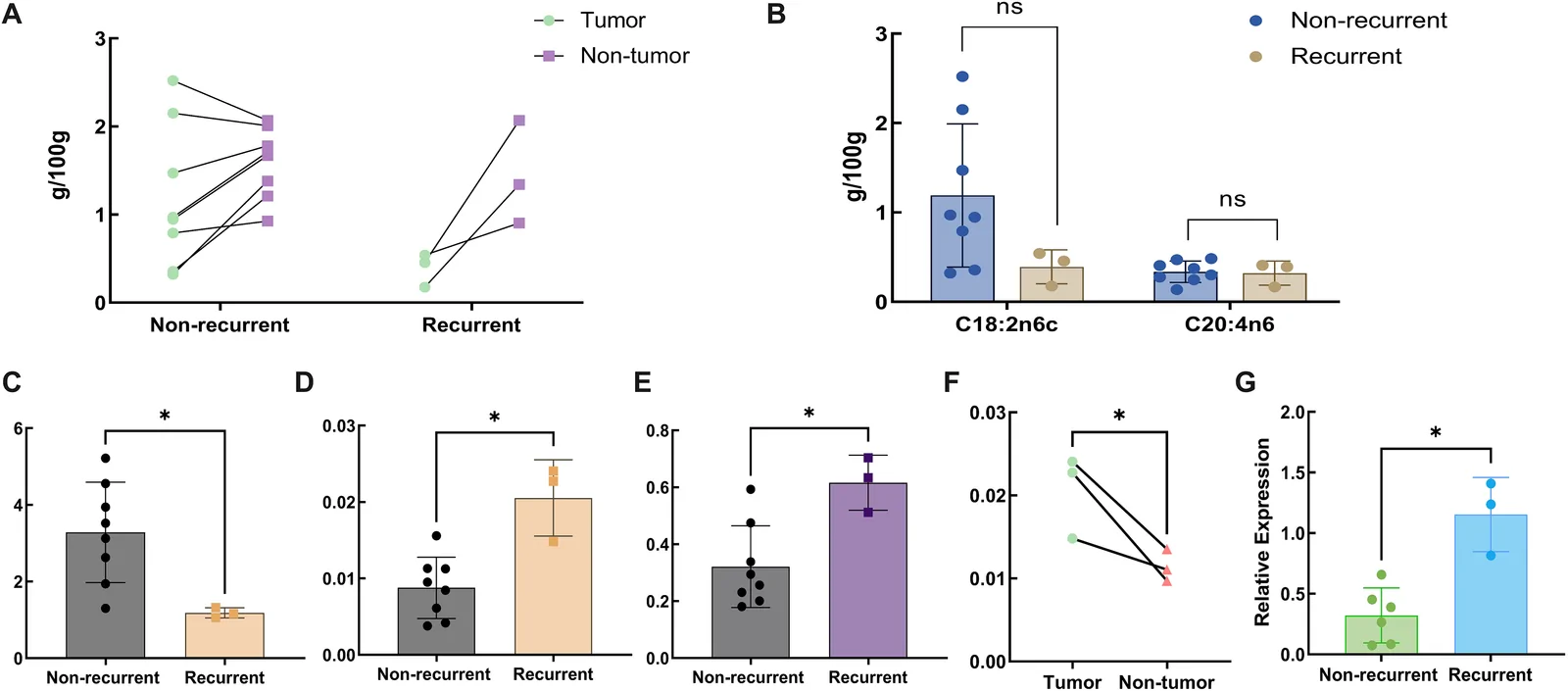
Recurrence-associated alterations in linoleic acid metabolism and n-6 PUFA desaturation indices in HCC. **(A)** Linoleic acid abundance in tumor and paired distal non-tumor tissues in the recurrent and non-recurrent groups; **(B)** Tumor linoleic acid and arachidonic acid levels in the non-recurrent vs recurrent groups; **(C)** Tumor LA/AA ratio in the non-recurrent vs recurrent groups; **(D)** Tumor estimated Δ6-desaturation index (GLA/LA; C18:3n-6/C18:2n-6) in the non-recurrent vs recurrent groups; **(E)** AA allocation relative to precursor pool (AA/(LA+GLA+DGLA)) in tumor tissues: recurrent vs non-recurrent; **(F)** Estimated Δ6-desaturation index in paired tumor vs distal non-tumor tissues within the recurrent group; **(G)** FADS2 mRNA expression in tumor tissues in the non-recurrent vs recurrent groups (qRT–PCR). (* *P* < 0.05).

Quantitative fatty acid profiling (37 analytes; LOD: 0.001 g/100 g) was performed on tumor tissues and matched distant non-tumor tissues from three patients with recurrent HCC and eight with non-recurrent HCC. In the recurrence group, linoleic acid levels were lower in tumor tissues than in their paired distant non-tumor tissues across all pairs, whereas this pattern was not observed in the non-recurrence group (**Figure 1A**). Comparative analysis of tumor tissues from the two clinical groups revealed no significant difference in absolute levels of linoleic acid (LA, C18:2n-6) or arachidonic acid (AA, C20:4n-6); however, the LA/AA ratio was significantly elevated in the non-recurrence group (**Figures 1B, C**).

The estimated Δ6-desaturation index (GLA/LA) was reduced in tumors from non-recurrent patients (**Figure 1D**). In contrast, the relative enrichment of arachidonic acid within the PUFA precursor pool, quantified by the AA/(LA + GLA + DGLA) ratio, was significantly elevated in tumor tissues from patients with recurrence (**Figure 1E**). By comparison, the elongation index (DGLA/GLA) and Δ5-desaturation index (AA/DGLA) showed no intergroup differences (**Supplementary Figures 2A, B**). Within the recurrent group, paired comparisons revealed that the estimated Δ6-desaturation index was significantly higher in tumor tissue than in matched distant normal tissue (**Figure 1F**), whereas DGLA/GLA and AA/DGLA remained stable (**Supplementary Figures 2C, D**). FADS2 mRNA expression was significantly higher in tumor tissues from the relapse group than in those from the no-recurrence group (**Figure 1G**). Moreover, in tumor tissues, the ratios AA/(LA + GLA + DGLA) and AA/total ω-6 PUFA were significantly lower in the non-recurrence group than in the recurrence group (**Supplementary Figures 2E, F**).

### 3.2 Single-cell profiling suggests recurrence-associated heterogeneity and malignant cell states in post-transplant HCC

**Figure 2:**
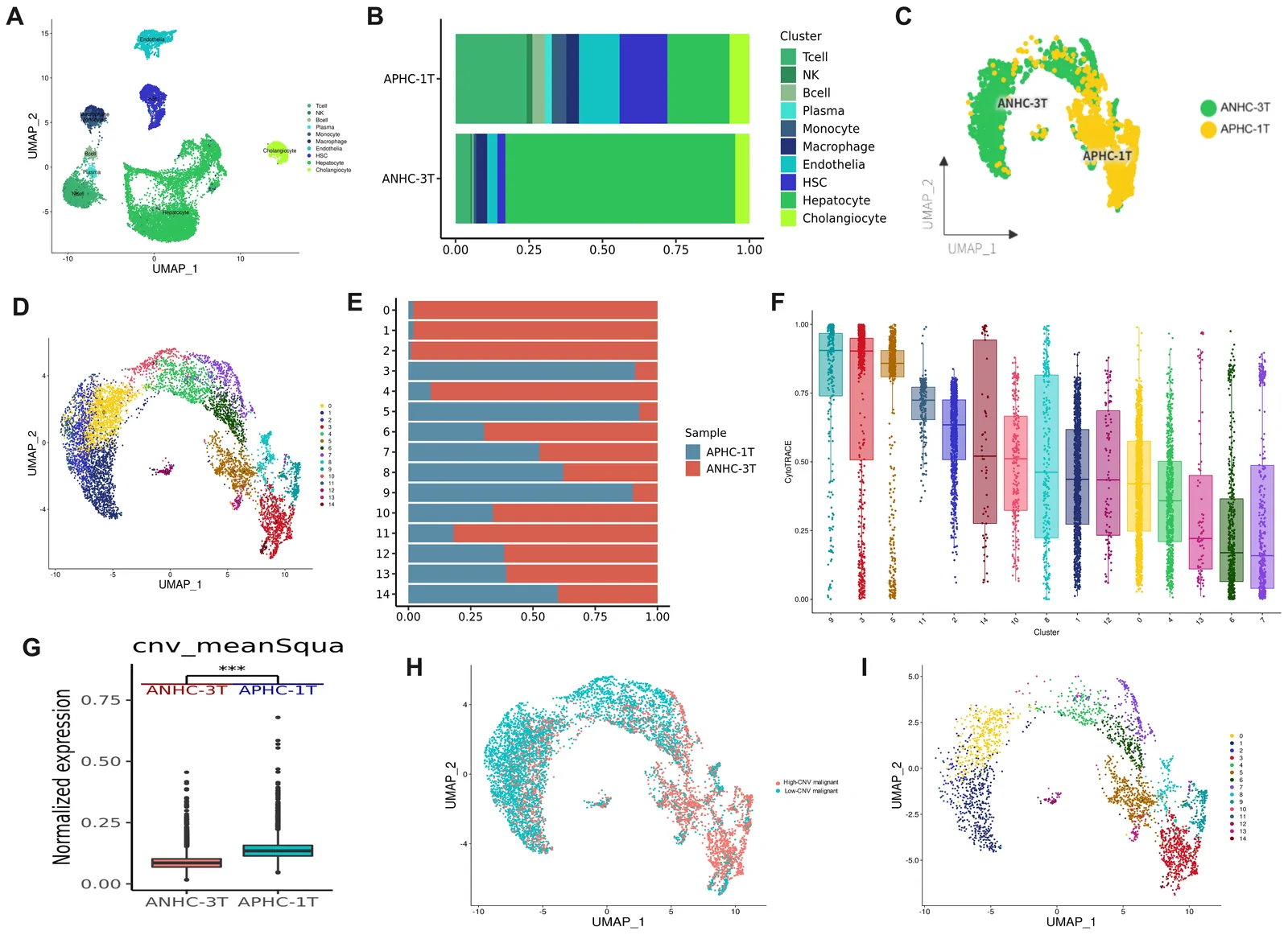
Single-cell transcriptomic atlas of recurrent and non-recurrent HCC. **(A)** Integrated UMAP of 14,463 cells colored by major lineages; **(B)** Cell type proportion shift between APHC-1T and ANHC-3T; **(C)** UMAP visualization of hepatocytes; **(D)** Unsupervised subclustering of hepatocytes; **(E)** Stacked bar plot showing sample distribution in 15 hepatocyte subclusters; **(F)** Comparison of CytoTRACE scores across clusters; **(G)** Copy number variation scores in representative non-recurrent and recurrent samples; **(H)** UMAP visualization of high-CNV malignant and low-CNV malignant compartments; **(I)** UMAP visualization of malignant cell subclusters.

To explore cellular features potentially associated with post-transplant HCC recurrence, we generated an exploratory snRNA-seq atlas using primary HCC tumors collected at the time of liver transplantation from recurrence-stratified LT-HCC cases. The APHC-1T case developed metastatic recurrence seven months after transplantation, whereas the ANHC-3T case remained recurrence-free for more than three years; the corresponding clinical information is provided in **Supplementary Table 5**. Single-cell transcriptomic profiling of 7,058 cells from relapsed tumors and 7,405 cells from non-relapsed tumors identified 10 major cell types and 15 transcriptionally distinct subclusters (**Figure 2A**; **Supplementary Figures 3A, B**). Annotation using canonical markers revealed nine populations: T, NK, B, and plasma cells; hepatocytes; hepatic stellate and cholangiocyte cells; monocytic cells; and endothelial cells (**Supplementary Figure 3C**). Relapsed tumors showed fewer hepatocytes and more immune cells than non-relapsed tumors (**Figure 2B**; **Supplementary Figure 3D**).

UMAP visualization across representative samples revealed spatially segregated hepatocyte distributions between relapse groups (**Figure 2C**). Unsupervised subclustering resolved hepatocytes into 15 transcriptionally defined subclusters (**Figure 2D**), with pronounced differences in subcluster abundance according to clinical outcome (**Figure 2E**). Specifically, subclusters 0, 1, 2, and 4 were significantly enriched in non-relapsed tumors, whereas subclusters 3, 5, and 9 were preferentially expanded in relapsed tumors. Notably, hepatocyte subclusters enriched in the recurrent tumor, particularly clusters 3, 5, and 9, showed significantly elevated CytoTRACE-inferred stemness scores (**Figure 2F**). At the sample level, the recurrent tumor also exhibited a higher overall copy number variation (CNV) burden than the non-recurrent tumor (*P* < 0.001; **Figure 2G**), consistent with a more pronounced malignancy-associated genomic alteration profile. Based on inferCNV-derived CNV burden, hepatocyte-lineage cells were further stratified into low-CNV malignant cells and high-CNV malignant cells (**Figure 2H**). Concordantly, high-CNV malignant cells were overwhelmingly localized within these recurrence-associated subpopulations, particularly subclusters 3 and 5 (**Figure 2I**).

### 3.3 Integrated analyses identify an ACSL3/EHHADH-associated fatty acid degradation program

To characterize potential recurrence-associated metabolic alterations, we performed three complementary comparisons: high-CNV malignant cells from the recurrent sample versus those from the non-recurrent sample, high-CNV malignant versus low-CNV malignant hepatocyte-lineage cells within the recurrent sample, and paired primary and recurrent HCC lesions from the external GSE249913 dataset.

**Figure 3:**
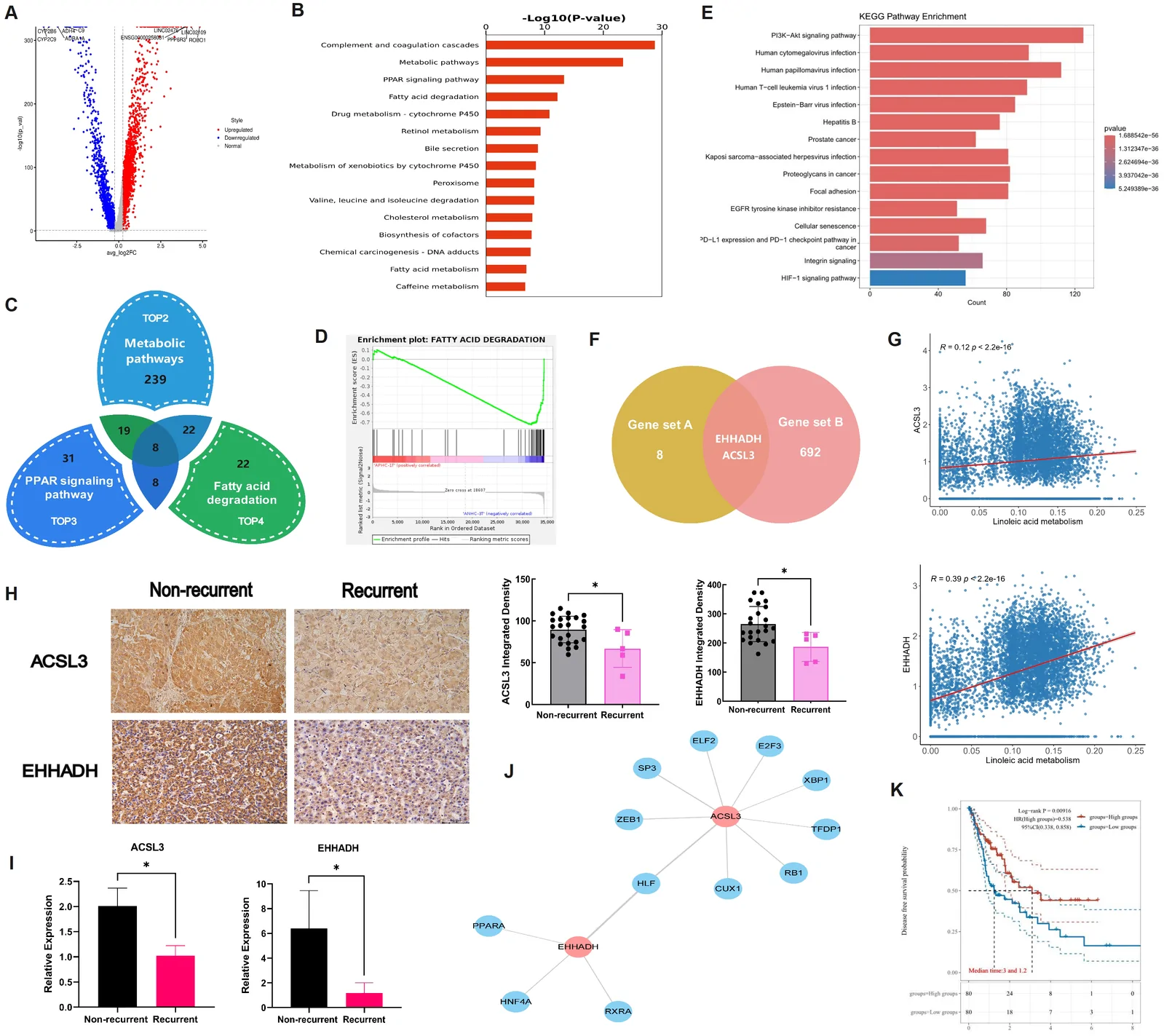
Integrated transcriptomic analyses, prognostic evaluation, and validation of genes associated with fatty acid degradation and linoleic acid metabolism. **(A)** Differential expression analysis of high-CNV malignant cells between ANHC-3T and APHC-1T; **(B)** KEGG enrichment analysis of genes downregulated in APHC-1T high-CNV malignant cells; **(C)** Venn diagram of the intersection of top enriched pathway genes; **(D)** Gene set enrichment analysis of fatty acid degradation pathway; **(E)** KEGG enrichment analysis of differentially regulated genes in recurrent lesions; **(F)** Venn diagram of the intersection of genes from Gene Set A and Gene Set B; **(G)** Correlation between linoleic acid metabolism (top: ACSL3; bottom: EHHADH); **(H)** Immunohistochemical staining of ACSL3 and EHHADH in tumor tissue; **(I)** qRT-PCR quantification of ACSL3 (left) and EHHADH (right) mRNA in tumor tissues; **(J)** Transcription factor enrichment analysis for ACSL3 and EHHADH; **(K)** DFS in TCGA-LIHC stratified by EHHADH expression in the FADS2-low subgroup. (*P < 0.05).

Differential expression analysis of high-CNV malignant cells between the recurrent and non-recurrent samples identified a distinct transcriptional profile in the recurrent samples (**Figure 3A**), with upregulated genes predominantly enriched in cell adhesion- and proteoglycan-related pathways (**Supplementary Figures 4A, B**). In contrast, downregulated genes in recurrent high-CNV malignant cells were mainly enriched in metabolic pathways, including PPAR signaling and fatty acid degradation (**Figure 3B**, **Supplementary Figure 4C**). The intersection of genes from the three highest-ranked metabolic pathways was used as a heuristic filtering strategy for downstream candidate screening, yielding an eight-gene candidate set designated as Gene Set A (**Figure 3C**). In the recurrent sample, ssGSEA analysis showed lower fatty acid metabolism scores in high-CNV malignant cells than in low-CNV malignant cells, largely reflecting reduced fatty acid degradation pathway activity (**Supplementary Figure 4D**). Consistently, GSEA comparing high-CNV malignant cells between the recurrent and non-recurrent samples demonstrated reduced fatty acid degradation in the recurrent sample (**Figure 3D**), which was further supported by ssGSEA-based pathway scoring (**Supplementary Figure 4E**).

In the GSE249913 dataset, differential expression analysis of seven paired primary and recurrent HCC lesions from liver transplant recipients identified genes significantly upregulated in recurrent lesions as Gene Set B. Enrichment analysis revealed that Gene Set B was significantly enriched in biological processes associated with immune cell activation, including the PI3K-Akt signaling pathway (**Figure 3E**, **Supplementary Figure 4F**). Intersection analysis of Gene Set A and Gene Set B yielded two candidate genes, ACSL3 and EHHADH (**Figure 3F**), implicated in metabolic remodeling during tumor recurrence.

At the single-nucleus transcriptomic level, ACSL3 and EHHADH were expressed at lower levels in high-CNV malignant cells from the recurrent sample than in those from the non-recurrent sample (**Supplementary Figure 4G**), and both genes were positively associated with the linoleic acid metabolism pathway score (**Figure 3G**). Consistently, tissue-level analyses showed higher ACSL3 and EHHADH protein (**Figure 3H**) and mRNA (**Figure 3I**) expression in tumors from the non-recurrent group compared with the recurrent group. Further transcription factor analysis identified HLF as a shared upstream regulator of both EHHADH and ACSL3 (**Figure 3J**), and HLF expression was significantly upregulated in non-recurrent high-CNV malignant cells (**Supplementary Figure 4H**).

To assess the clinical relevance of these LA metabolism-related genes in a broader HCC context, we examined their prognostic associations in TCGA-LIHC, a non-transplant HCC cohort. High EHHADH expression was associated with prolonged DFS in the FADS2-low subgroup (*P* = 0.009; **Figure 3K**), but did not significantly stratify OS or DFS in the FADS2-high subgroup (**Supplementary Figure 5A**). High ACSL3 expression was associated with shorter OS across both FADS2-defined subgroups (**Supplementary Figure 5B**), without significant DFS stratification. In addition, within the EHHADH-high subgroup, ACSL3 expression did not provide additional prognostic stratification for either OS or DFS (**Supplementary Figure 5C**).

### 3.4 Single-cell analysis links linoleic acid metabolic features to Treg-associated immunosuppressive remodeling

**Figure 4:**
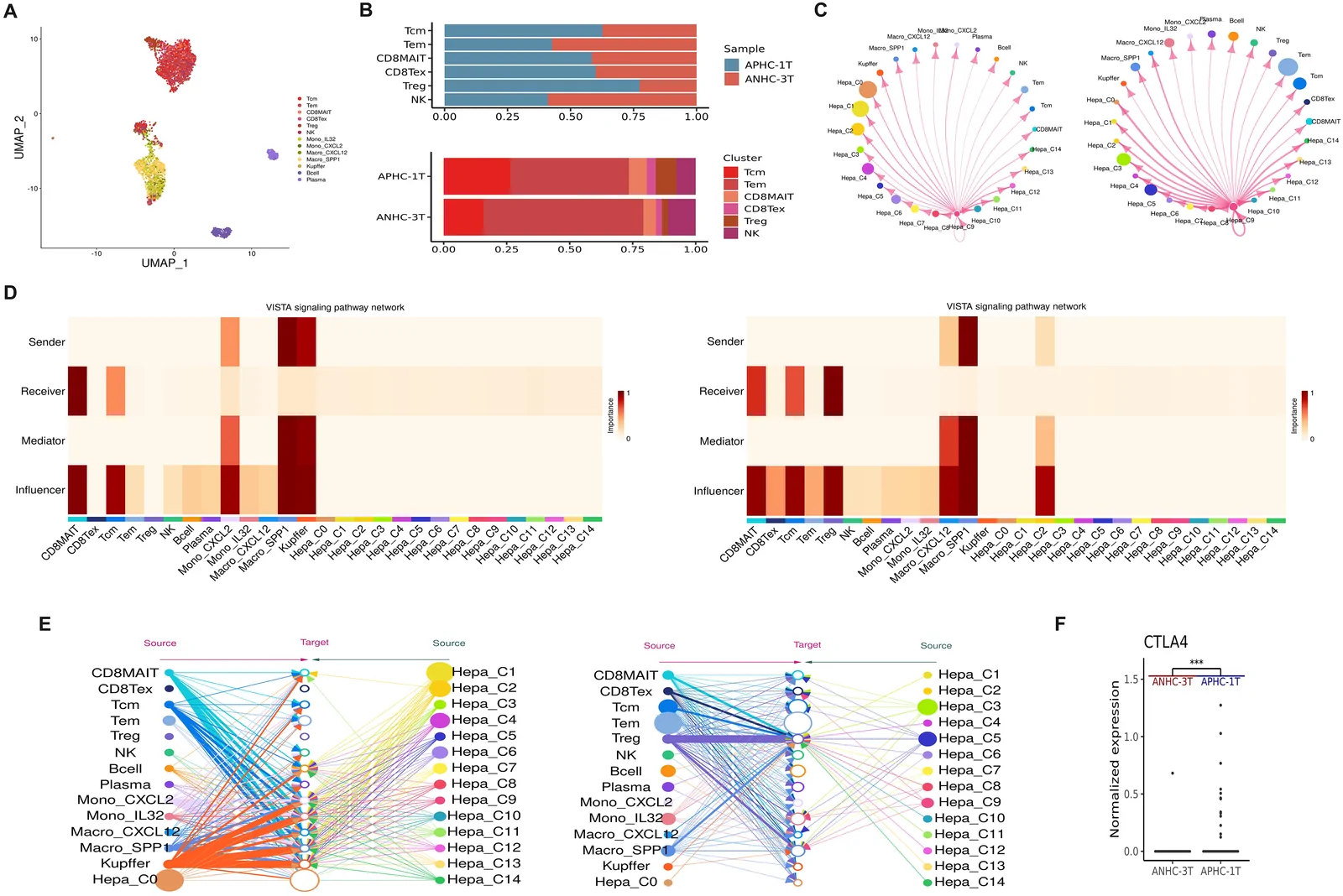
Non-recurrent vs recurrent HCC: immune atlas, cell–cell communication. **(A)** UMAP visualization of 13 immune-related molecular subtypes; **(B)** Proportions of T-cell subclusters across samples; **(C)** CellChat2-predicted intercellular communication network centered on malignant cluster 9 (left: Non-recurrent; right: Recurrent); **(D)** Pathway-level VISTA signaling network inferred by CellChat2 (top: Non-recurrent; bottom: Recurrent); **(E)** Hierarchical analysis of CD80–CTLA4 interaction strength (left: Non-recurrent; right: Recurrent); **(F)** Differential expression of CTLA4 in non-recurrent versus recurrent tumors. (*P < 0.05, **P < 0.01, ***P < 0.001).

Single-cell RNA sequencing identified six major immune lineages (**Supplementary Figure 6A**) and, via unsupervised subclustering, 13 transcriptionally and functionally distinct immune states (**Figure 4A**). Macrophages showed the largest compositional change across samples (**Supplementary Figure 6B**). The non-recurrent sample had expanded Kupffer cells (**Supplementary Figure 6C**) and fewer Treg cells (**Figure 4B**), while B and plasma cell proportions were similar between the recurrent and non-recurrent groups (**Supplementary Figure 6D**). Focusing on the high-CNV malignant subclusters with the strongest stemness signatures (clusters 3, 5, and 9), we applied CellChat2 to reconstruct intercellular communication networks. In the recurrent sample, tumor-to-Treg signaling increased across all three subclusters, with cluster 9 acting as the main hub (**Figure 4C**, **Supplementary Figures 6E, F**).

At the pathway level, CellChat2 analysis showed differences in VISTA-related communication between the non-recurrent and recurrent samples. In the non-recurrent sample, VISTA signaling was mainly inferred from Kupffer cells and SPP1^+^ macrophages toward CD8^+^ MAIT cells, whereas in the recurrent sample, SPP1^+^ macrophages showed stronger inferred communication toward Treg cells (**Figure 4D**).

Additional checkpoint/co-regulatory pathways were further examined. PVR, NECTIN, CD80, and CD86 signaling showed distinct communication patterns between the non-recurrent and recurrent samples (**Figure 4E**, **Supplementary Figure 7**). In the recurrent sample, Treg cells showed more evident inferred involvement in PVR-, CD80-, and CD86-related networks, particularly in receiver, mediator, or influencer roles. PVR and NECTIN signaling also involved hepatocyte-lineage compartments, including selected high-CNV malignant hepatocyte-like clusters Hepa_C3, Hepa_C5, and Hepa_C9. Consistently, hierarchical analysis of CD80-CD28 interaction strength further showed enhanced Treg-related communication in the recurrent sample (**Supplementary Figure 6G**).

Predicted Treg-directed checkpoint and co-regulatory ligand–receptor interactions exhibited sample-specific patterns, including CTLA4- and TIGIT-associated inhibitory axes in the recurrent sample (**Supplementary Figure 6H**). At the gene-expression level, PDCD1 showed low expression, whereas CTLA4 expression was higher in the recurrent sample (**Figure 4F**). CellChat2-based signaling role analysis of IL1, IL6, IL10, and SPP1 signaling pathways was performed in ANHC-3T and APHC-1T. Among the four inferred roles assessed, inter-sample differences were mainly observed in receiver and influencer roles across immune and hepatocyte-lineage compartments (**Supplementary Figure 8**).

### 3.5 Clinical and tissue-level analyses reveal recurrence-associated immune features

To further substantiate the immune-related findings from the single-nucleus RNA sequencing analysis, we evaluated tumor immune-related markers, CD4^+^FOXP3^+^ Treg infiltration, circulating lymphocyte subsets, and postoperative serum cytokine levels in recurrent and non-recurrent HCC patients.

**Figure 5:**
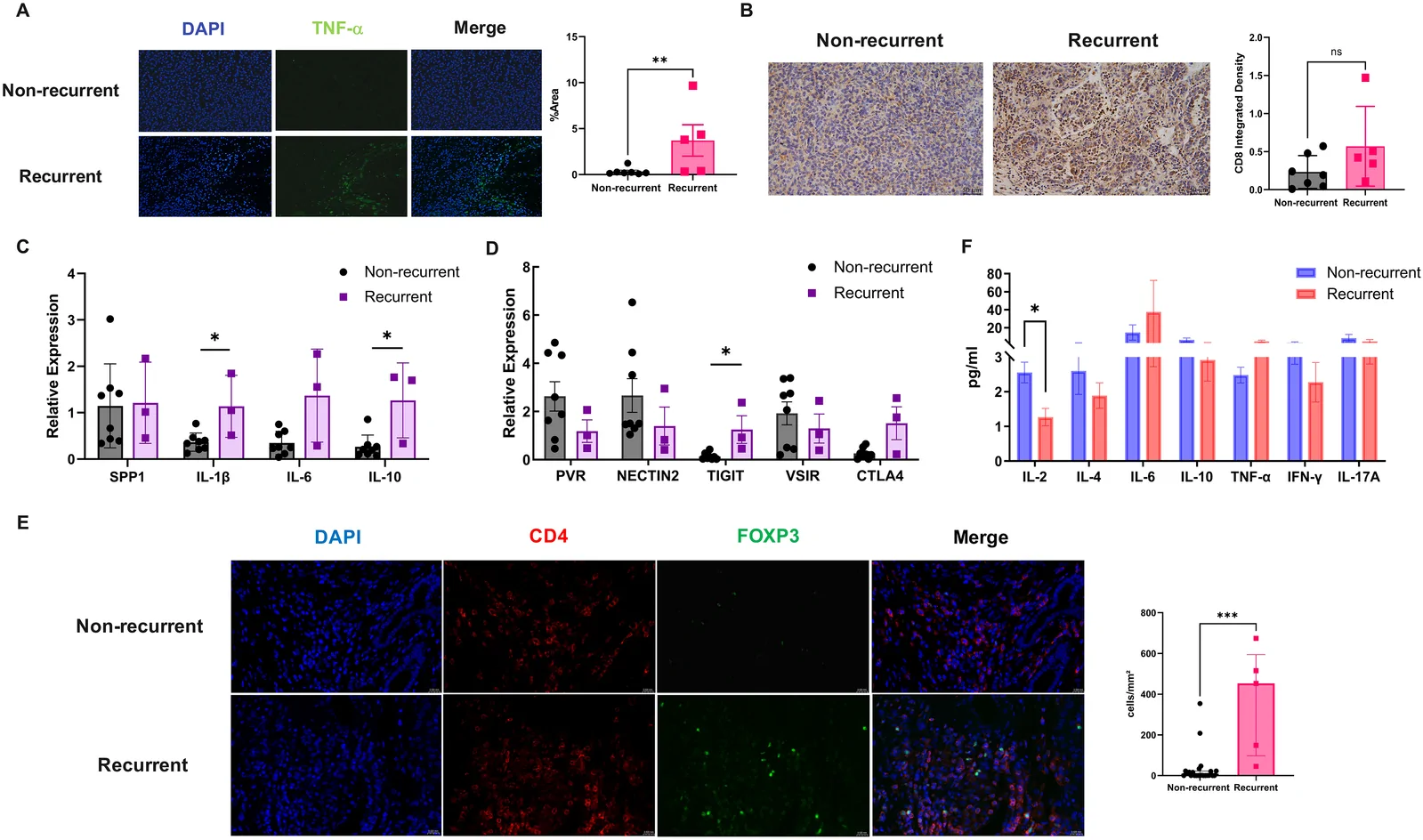
Recurrence-associated tumor-tissue and serum immune features in post-transplant HCC. **(A)** TNF-a immunofluorescence staining in tumor tissue sections; **(B)** CD8 immunohistochemical staining in tumor tissue sections; **(C)** qRT-PCR detection of inflammatory and immune-related transcripts in tumor tissues. **(D)** qRT-PCR analysis of PVR, NECTIN2, VSIR, TIGIT, and CTLA4 expression in tumor tissues (mean ± SEM); **(E)** CD4/FOXP3 dual immunofluorescence staining for CD4+FOXP3+ Treg infiltration in tumor tissue sections (median with IQR); **(F)** Flow cytometry-based detection of serum cytokines at 12 months post-transplantation. (*P < 0.05, **P < 0.01, ***P < 0.001).

Tumor tissue analysis showed significantly elevated IL-1β, IL-10, and TNF-α levels in recurrent tumors, whereas IL-6, SPP1, and CD8 expression did not differ significantly between groups (**Figures 5A–C**). qRT-PCR analysis of immune checkpoint and co-regulatory molecules implicated by the single-cell communication analysis further showed selective upregulation of TIGIT in recurrent tumors, while PVR, NECTIN2, VSIR, and CTLA4 did not differ significantly between groups (**Figure 5D**). CD4/FOXP3 dual immunofluorescence further demonstrated significantly increased CD4^+^FOXP3^+^ Treg infiltration in recurrent tumors (**Figure 5E**). Preoperative peripheral blood lymphocyte subset analysis showed a significantly higher absolute CD8^+^ T-cell count in the non-recurrent group, whereas the recurrent group exhibited a significantly higher NK-cell proportion (**Table 1**). In contrast to the preoperative differences, peripheral blood lymphocyte subsets at 1 month after transplantation were broadly comparable between the recurrent and non-recurrent groups (**Supplementary Table 6**). At 12 months post-surgery, serum cytokine analysis revealed reduced IL-2 levels in the recurrent group (**Figure 5F**). To account for immunosuppression intensity as a potential confounder of immune phenotypes, tacrolimus trough concentrations were summarized across predefined post-transplant time windows and compared by recurrence status, with no significant differences observed within the first 24 months after transplantation (**Supplementary Table 7**).

**Table 1:** Preoperative peripheral blood lymphocyte subsets in non-recurrent and recurrent groups.

Lymphocyte subset	Proportion (%)		P - value	Absolute count (cells/µL)		P - value
	Non-recurrent group	Recurrent group		Non-recurrent group	Recurrent group	
Lymphocytes	11.72 (7.02-16.61)	6.140 (4.00-23.74)	0.591	1212 (846.5-1638)	619.0 (588.0-1273)	0.197
T cells	72.24 (69.76-83.65)	63.50 (58.42-81.37)	0.244	925.5 (718.8-1179)	374.0 (362.0-1036)	0.197
CD4^+^ T	47.08 (35.60-51.83)	41.93 (33.37-50.84)	0.677	506.5 (408.0-814.0)	247.0 (207.0-647.0)	0.162
CD8^+^ T	22.87 (17.56-32.11)	12.53 (10.73-20.73)	0.121	247.5 (176.8-458.0)	128.0 (63.00-159.0)	0.032*
B cells	11.65 (5.13-21.71)	11.57 (5.24-22.44)	0.999	138.0 (46.00-294.5)	68.00 (66.00-132.0)	0.509
NK cells	6.500 (4.39-12.32)	18.68 (12.81-25.23)	0.032*	81.50 (45.00-188.8)	145.0 (110.0-165.0)	0.421

Lymphocytes (%) represents the percentage of lymphocytes among total leukocytes. *P < 0.05.

### 3.6 LA-BSA exposure alters redox status and immunometabolism-related gene expression in HCC cells

**Figure 6:**
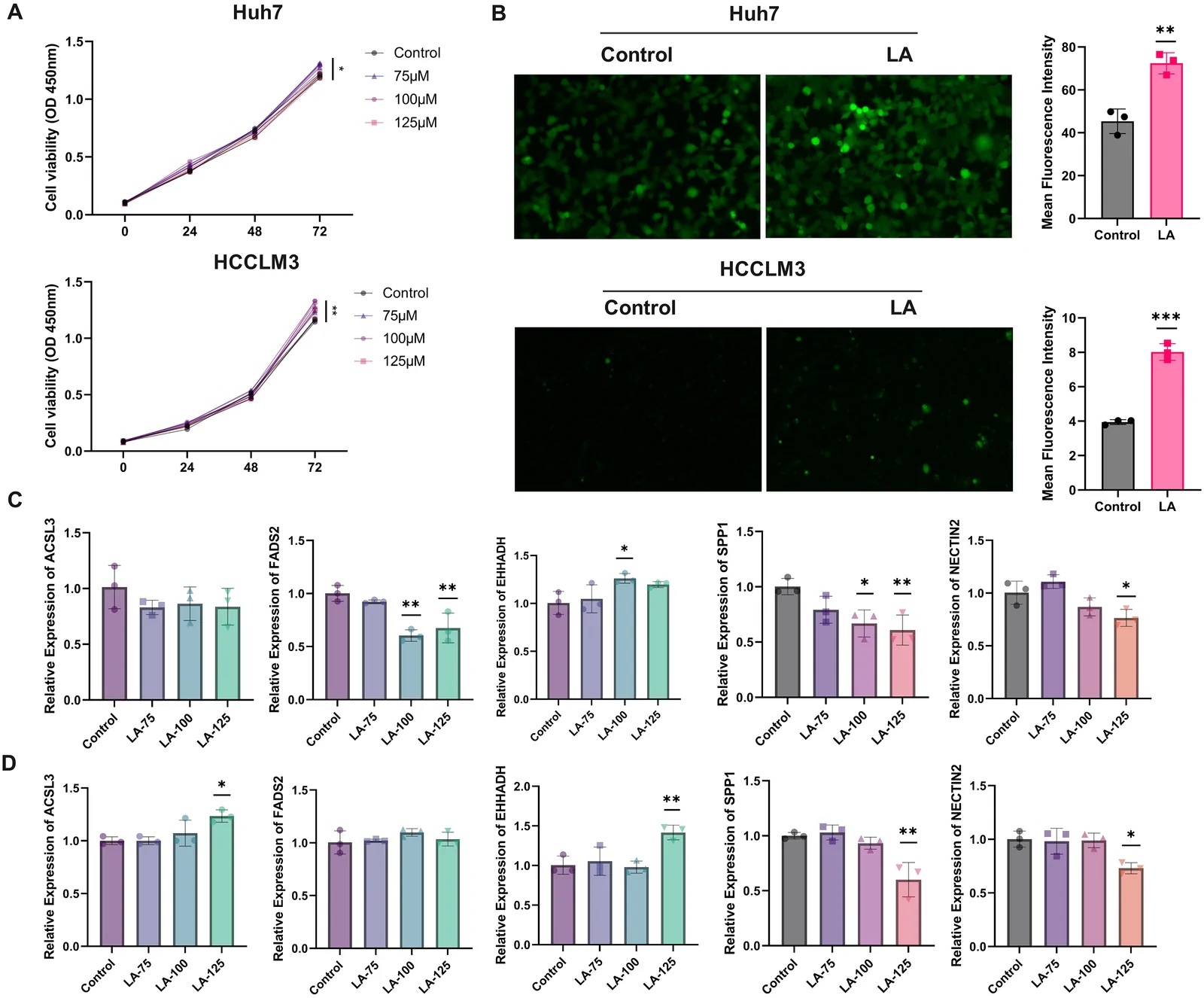
Cellular responses to linoleic acid exposure in Huh7 and HCCLM3 hepatocellular carcinoma cell lines. **(A)** CCK-8 assay of cell viability following linoleic acid treatment; **(B)** Intracellular ROS levels after linoleic acid exposure; **(C, D)** Relative mRNA expression of ACSL3, FADS2, EHHADH, SPP1, and NECTIN2 in Huh7 **(C)** and HCCLM3 **(D)** cells treated with different concentrations of linoleic acid. (*P < 0.05, **P < 0.01, ***P < 0.001).

To further evaluate the responses of HCC cells under lower and more interpretable LA exposure conditions, HCCLM3 and Huh7 cells were treated with 75, 100, or 125 μM LA-BSA. CCK-8 assays showed increased metabolic viability signals in HCC cells treated with 75 and 100 μM LA-BSA, whereas no significant change was observed following treatment with 125 μM LA-BSA (**Figure 6A**). ROS assays showed that LA-BSA treatment increased intracellular ROS levels in HCC cells (**Figure 6B**). qRT-PCR was performed to assess the expression of lipid metabolism–related genes and immunomodulatory factors following LA-BSA treatment. In Huh7 cells, ACSL3 expression remained unchanged, whereas EHHADH expression increased at 100 μM LA-BSA. FADS2 and SPP1 expression decreased at both 100 and 125 μM LA-BSA, while NECTIN2 expression was reduced only at 125 μM (**Figure 6C**). In HCCLM3 cells, treatment with 125 μM LA-BSA increased ACSL3 and EHHADH expression but decreased SPP1 and NECTIN2 expression (**Figure 6D**).

## 4 Discussion

In this study, we integrated single-cell tumor–immune communication analysis, quantitative fatty acid profiling, and external cohort validation to characterize a recurrence-associated immunometabolic framework. Given the current paucity of single-cell studies in post-transplant HCC recurrence, this multi-layered approach provides a more integrated perspective on recurrence-linked immunometabolic remodeling. Specifically, post-transplant recurrence was associated with an intratumoral LA metabolic imbalance characterized by a higher FADS2-related estimated Δ6-desaturation index and an attenuated EHHADH-associated oxidative catabolism signature, with ACSL3 expression potentially reflecting upstream fatty-acid activation-related features. This metabolic pattern was accompanied by enhanced Treg-associated immunosuppressive features. Together, these findings indicate that post-transplant HCC recurrence is associated with coordinated immunometabolic remodeling characterized by altered LA metabolic partitioning and an immunosuppressive tumor microenvironment. This integrated immunometabolic pattern provides a biological basis for understanding recurrence-associated metabolicimmune remodeling in post-transplant HCC.

Previous studies have functionally implicated FADS2-mediated Δ6-desaturation as an important upstream regulatory node in n-6 PUFA metabolism and tumor lipid remodeling (22–24). In this study, our data indicate that the metabolic distinction between recurrent and non-recurrent tumors is not a uniform alteration of the entire LA-to-AA biosynthetic cascade but is instead localized primarily to the Δ6-desaturation–related allocation step. Specifically, non-recurrent tumors were characterized by a higher intratumoral LA/AA ratio together with a lower estimated Δ6-desaturation index than recurrent tumors, whereas the elongation and Δ5-desaturation indices were largely comparable between groups. Notably, the LA/AA ratio and the GLA/LA ratio capture distinct aspects of pathway dynamics: the former reflects the overall balance between substrate and terminal product, whereas the latter more directly reflects relative metabolic allocation at the FADS2-associated Δ6-desaturation step (25, 26). Accordingly, a lower estimated Δ6-desaturation index in non-recurrent tumors should not be taken to indicate a proportionate reduction in AA abundance. Instead, downstream AA homeostasis may be maintained through compensatory mechanisms, including alternative substrate supply, exogenous PUFA uptake, membrane phospholipid remodeling, and phospholipase-mediated AA release (27, 28). Taken together, these findings support a model in which altered LA allocation at the FADS2-associated upstream branch, rather than generalized attenuation of downstream AA biosynthesis, may represent a key metabolic distinction between recurrent and non-recurrent tumors.

Another key metabolic pathway for LA involves ACSL3-mediated activation to LA-CoA, followed by entry into the EHHADH-associated peroxisomal β-oxidation pathway (29). In this pathway, ACOX-dependent oxidation generates H_2_O_2_, whereas EHHADH functions as a core bifunctional enzyme catalyzing subsequent β-oxidation steps, thereby linking LA oxidative catabolism to redox metabolic regulation (30, 31). Studies have shown that excessive ROS in tumor models compromises FOXP3 protein stability and impairs Treg suppressive function (32). Consistent with this, we observed a significant increase in intracellular ROS levels in HCC cells following LA-BSA exposure *in vitro*, indicating enhanced oxidative stress. In this context, greater engagement of EHHADH-associated oxidative disposal provides a plausible metabolic explanation for ROS accumulation and may impose additional antioxidant demand (33). Such redox pressure may, in turn, influence immunosuppressive signaling and contribute to the remodeling of tumor–Treg crosstalk through metabolically conditioned signaling.

From an immune-regulatory perspective, single-cell communication analysis indicated that recurrence-associated metabolic remodeling was accompanied by alterations in checkpoint-related and co-regulatory interactions. VISTA is an immune checkpoint prominently expressed in myeloid compartments and implicated in maintaining T-cell quiescence, peripheral tolerance, and suppression of T-cell activation within the tumor microenvironment (34). Consistent with previous reports, our expression analysis showed that VSIR/VISTA was more evident in myeloid-lineage populations than in Tregs. This expression pattern suggests that VISTA-related communication may primarily reflect myeloid-associated immunoregulation rather than Treg-intrinsic VISTA activity (35). Importantly, the recurrence-associated immune communication pattern was not limited to VISTA signaling. Additional analyses of PVR, NECTIN2, CD80, and CD86 signaling suggested a recurrence-associated shift in checkpoint/co-regulatory communication. The non-recurrent sample showed inferred myeloid cell interactions mainly involving CD8^+^/MAIT cells, whereas the recurrent sample showed more prominent myeloid cell–Treg-associated communication, consistent with a more immunoregulatory immune contexture (36). Accordingly, CD80-related communication was interpreted within the broader CD28/CTLA4–CD80/CD86 co-regulatory framework, rather than as an isolated CD80 signal (37). This interpretation was further supported by higher CTLA4 expression in the recurrent sample, whereas weak PDCD1 expression and limited PDCD1-related communication did not favor a PD-1-centered interpretation of the observed immune remodeling. In the post-transplant immunosuppressed setting, these exploratory findings suggest that myeloid–Treg coupling and checkpoint/co-regulatory interactions may contribute to a recurrence-associated immunosuppressive milieu, although further validation is required.

Although these single-cell observations alone cannot establish population-level immune circuitry, their concordance with tissue-level findings supports their relevance to the recurrence-associated immunometabolic features observed in this study. At the tumor-tissue level, the combined increase in IL-10 expression, CD4^+^FOXP3^+^ Treg infiltration, and TIGIT expression in recurrent tumor tissues suggests that recurrence is associated with a local immunoregulatory state rather than merely a nonspecific inflammatory response. This tissue-level pattern supports the single-cell inference that Treg-related inhibitory communication, particularly TIGIT-associated signaling, may contribute to recurrence-associated immune remodeling (38). Although PVR, NECTIN2, VSIR, and CTLA4 did not differ significantly at the bulk tumor-tissue level, their single-cell communication patterns were cell-type-specific. VSIR-related signaling was mainly linked to myeloid-lineage populations (36), whereas PVR- and NECTIN2-related interactions involved monocyte and macrophage subsets as well as high-CNV and low-CNV hepatocyte-lineage compartments. Thus, these molecules may be more informative at the cell-type-resolved communication level than at the whole-tissue transcript level. Notably, although pre-transplant peripheral cytotoxic immune composition differed between the two groups, neither early postoperative peripheral immune status nor tacrolimus exposure showed corresponding differences. This further suggests that recurrence-associated tumor-immune features may depend more on the local immune microenvironment of the primary tumor than on postoperative systemic immune status or differential immunosuppressive exposure (39).

To further investigate whether metabolic gene expression patterns induced by exogenous LA loading are accompanied by changes in immune-related gene expression, HCC cells were treated with different concentrations of LA-BSA. LA exposure altered the expression of lipid metabolism–related genes and increased intracellular ROS accumulation, indicating changes in the metabolic and redox state of HCC cells. Notably, LA treatment, particularly at higher concentrations, was accompanied by reduced SPP1 and NECTIN2 expression. SPP1 has been implicated in SPP1^+^ macrophage-associated intercellular communication, CD8^+^ T-cell dysfunction or exclusion, and tumor immune evasion (40), whereas NECTIN2 interacts with both activating and inhibitory immune receptors and may contribute to tumor immune evasion when inhibitory signaling predominates (41). These findings provide supportive evidence that LA-related metabolic perturbation may be accompanied by changes in tumor-cell immunomodulatory features, although their functional immune consequences require further investigation.

Although previous studies have linked ACSL3 overexpression to poor prognosis in HCC (42), our TCGA-LIHC analysis supports a more context-dependent interpretation. ACSL3 was mainly associated with OS rather than DFS, and its prognostic stratification was not evident within the EHHADH-high subgroup. By contrast, the DFS-related association of EHHADH appeared more evident in the FADS2-low subgroup. These observations are broadly in line with our transplant cohort findings, which suggested a lower FADS2-related desaturation signature and relatively preserved EHHADH-associated fatty-acid oxidative catabolism in the non-recurrent sample. Thus, the TCGA-LIHC results provide indirect support for the clinical relevance of LA metabolism-related gene states in HCC, while indicating that ACSL3 should be interpreted within the broader FADS2/EHHADH-associated metabolic background rather than as an isolated recurrence-related marker. Nevertheless, because TCGA-LIHC is not a liver transplantation-specific cohort, these findings should be considered exploratory.

From a translational perspective, our findings suggest the potential value of risk stratification, differentiated management, and nutrition-oriented intervention in HCC after liver transplantation. Recurrence-related metabolic–immune features centered on tumor linoleic acid reprogramming and Treg-associated checkpoint remodeling may help identify patients at higher risk of recurrence. Such patients may benefit from intensified monitoring of immunosuppressant levels, peripheral lymphocyte subsets, and circulating immunometabolic mediators. Moderate linoleic acid supplementation may also represent a potential strategy to reduce recurrence risk, although its safety and clinical benefit require prospective validation.

This study has several limitations. First, despite a 5-year follow-up, the number of post-transplant HCC recurrence events available for analysis remained limited. Compared with conventional hepatectomy for HCC, liver transplantation is performed in a smaller HCC patient population and is associated with a lower recurrence rate. Therefore, obtaining sufficient recurrent samples at the single-nucleus transcriptomic level would require upfront sequencing of a large number of LT-HCC cases followed by long-term outcome ascertainment. However, the substantial cost of snRNA-seq and downstream bioinformatic analysis makes such a strategy difficult to implement, which explains why single-cell or single-nucleus studies of post-transplant HCC recurrence remain rare. In addition, delayed recurrence ascertainment and stringent technical requirements for sequencing-qualified samples further limited the construction of a recurrence-stratified cohort. Accordingly, the snRNA-seq findings should be interpreted primarily as exploratory and hypothesis-generating evidence rather than independent quantitative or causal conclusions. Another methodological limitation is that the metabolism-related pathways used to construct Gene Set A have partially overlapping gene annotations. Therefore, the resulting gene overlap should not be interpreted as evidence of multiple independent biological signals.

To mitigate uncertainty arising from small sample sizes, we prioritized robust nonparametric methods, analyzed biologically informed within-sample ratios to minimize inter-individual variability, and validated key associations in independent external cohorts. Despite these limitations, consistent metabolic and immune-related patterns were observed across the clinical cohort, tumor tissue analyses, and independent external datasets, providing supportive evidence for the main findings of this study. Future studies using larger, multicenter LT-HCC cohorts and prospective biobanks are needed to validate the associations between LA metabolic reprogramming, immunometabolic remodeling, and post-transplant recurrence risk. Mechanistic studies incorporating functional perturbation, stable isotope tracing, and *in vivo* models of HCC recurrence will be required to determine whether LA metabolic pathways play a causal role in tumor recurrence and the remodeling of the immunosuppressive tumor microenvironment.

## 5 Conclusion

This study integrated intratumoral single-cell profiling with quantitative fatty acid analysis and validation in independent cohorts and public datasets to delineate a recurrence-associated immunometabolic landscape. Collectively, our findings suggest that an imbalance in LA metabolic partitioning, accompanied by Treg-associated immunosuppressive remodeling, represents a characteristic immunometabolic feature of post-transplant HCC recurrence. These findings provide a rationale for future studies aimed at improving recurrence risk stratification and exploring the translational potential of immunometabolic biomarkers in post-transplant HCC.

## Data Availability

The datasets presented in this study can be found in online repositories. The names of the repository/repositories and accession number(s) can be found below: https://www.ncbi.nlm.nih.gov/, GSE304188.
