# Supplementary material for: Linoleic acid metabolic reprogramming is linked to immunometabolic remodeling and post-transplant recurrence risk in hepatocellular carcinoma
Source: Front Immunol. 2026 Aug 11;17:1862165. doi: 10.3389/fimmu.2026.1862165 (PMC13503599; doi:10.3389/fimmu.2026.1862165)
Supplement: Supplementary file 1 [file DataSheet1.docx]

**Supplementary Figure**

**Supplementary Figure 1.** Patient selection flow for the liver transplantation HCC cohort.

**Supplementary Figure 2.** Downstream n-6 PUFA pathway indices and arachidonic acid allocation metrics.

**Supplementary Figure 3.** Subclustering, annotation, and compositional differences between ANHC-3T and APHC-1T.

**Supplementary Figure 4.** Functional enrichment and fatty acid degradation analyses associated with high-CNV malignant cells and recurrence.

**Supplementary Figure 5.** Complementary stratified survival analyses of LA metabolism-related genes in the TCGA-LIHC cohort.

**Supplementary Figure 6.** Immune-cell composition and intercellular communication patterns.

**Supplementary Figure 7.** Additional inferred immune checkpoint and co-regulatory signaling networks.

**Supplementary Figure 8.** Signaling role analysis of cytokine-related pathways in non-recurrent and recurrent samples.

**Supplementary Figure 1.** Patient selection flow for the liver transplantation HCC cohort.

**Supplementary Figure 2.** Downstream n-6 PUFA pathway indices and arachidonic acid allocation metrics.

(A) Elongation index (DGLA/GLA) in tumor tissues: recurrent vs non-recurrent; (B) Δ5 desaturation index (AA/DGLA) in tumor tissues: recurrent vs non-recurrent; (C) Elongation index (DGLA/GLA) in paired tissues within the recurrent group: tumor vs distal non-tumor; (D) Δ5 desaturation index (AA/DGLA) in paired tissues within the recurrent group: tumor vs distal non-tumor; (E) AA fraction of the ω-6 PUFA pool (AA/total ω-6 PUFA) in tumor tissues: recurrent vs non-recurrent; (F) (GLA + DGLA + AA)/LA ratio in tumor tissues: recurrent vs non-recurrent groups. (* *P* < 0.05)

**Supplementary Figure 3.** Subclustering, annotation, and compositional differences between ANHC-3T and APHC-1T.

(A) Unsupervised subclustering identifies 15 transcriptionally distinct cell subclusters; (B) UMAP visualization of the single-nucleus datasets (Left: ANHC-3T; Right: APHC-1T); (C) Marker gene expression for defined subclusters; (D) Cell type proportion shift between APHC-1T and ANHC-3T.

**Supplementary Figure 4.** Functional enrichment and fatty acid degradation analyses associated with high-CNV malignant cells and recurrence.

(A) GO enrichment analysis of upregulated genes in APHC-1T high-CNV malignant cells ; (B) KEGG pathway enrichment analysis of upregulated genes in APHC-1T high-CNV malignant cells; (C) GO enrichment analysis of downregulated genes in APHC-1T high-CNV malignant cells; (D) Single-sample GSEA of fatty acid degradation by cell type; (E) Single-sample GSEA of fatty acid degradation between samples; (F) GO enrichment analysis of differentially regulated genes in recurrent lesions; (G) Gene expression in high-CNV malignant cells (Left: ACSL3; Right: EHHADH); (H) HLF expression levels in high-CNV malignant cells across sample groups. (*** *P* < 0.001)

**Supplementary Figure 5.** Complementary stratified survival analyses of LA metabolism-related genes in the TCGA-LIHC cohort.

(A) Kaplan-Meier curves for OS (left) and DFS (right) stratified by EHHADH expression in the FADS2-high subgroup; (B) Kaplan-Meier curves for OS stratified by ACSL3 expression in the FADS2-low subgroup (upper) and FADS2-high subgroup (lower); (C) Kaplan-Meier curves for OS (left) and DFS (right) stratified by ACSL3 expression in the EHHADH-high subgroup.

**Supplementary Figure 6.** Immune-cell composition and intercellular communication patterns.

(A) UMAP visualization of the six major immune lineages; (B) Relative proportions of the six major immune lineages; (C) Proportions of monocytes and macrophages across samples; (D) Proportions of B cells and plasma cells across samples; (E) CellChat2-predicted intercellular communication network centered on high-CNV malignant cluster 3 (left: non-recurrent; right: recurrent); (F) CellChat2-predicted intercellular communication network centered on high-CNV malignant cluster 5 (left: non-recurrent; right: recurrent); (G) Hierarchical analysis of CD80-CD28 interaction strength (left: non-recurrent; right: recurrent); (H) Predicted Treg-directed checkpoint/co-regulatory ligand-receptor interactions in ANHC-3T and APHC-1T.

**Supplementary Figure 7.** Additional inferred immune checkpoint and co-regulatory signaling networks.

(A) PVR signaling pathway network; (B) NECTIN signaling pathway network; (C) CD80 signaling pathway network; (D) CD86 signaling pathway network; For each panel, the left heatmap represents the non-recurrent sample, and the right heatmap represents the recurrent sample.

**Supplementary Figure 8**. Signaling role analysis of cytokine-related pathways across non-recurrent and recurrent samples.

(A) IL1-related signaling roles in non-recurrent and recurrent samples (Left: non-recurrent; right: recurrent); (B) IL6-related signaling roles in non-recurrent and recurrent samples (Left: non-recurrent; right: recurrent); (C) IL10-related signaling roles in the recurrent sample; (D) SPP1-related signaling roles in the recurrent sample. For panels C and D, the corresponding pathways were not inferred in the non-recurrent sample after CellChat2 filtering.
