# Supplementary material for: Linoleic acid metabolic reprogramming is linked to immunometabolic remodeling and post-transplant recurrence risk in hepatocellular carcinoma
Source: Front Immunol. 2026 Aug 11;17:1862165. doi: 10.3389/fimmu.2026.1862165 (PMC13503599; doi:10.3389/fimmu.2026.1862165)
Supplement: Supplementary file 2 [file Table1.docx]

**Supplementary Tables**

**Supplementary Table 1.** Comparative demographic, clinical, and tumor pathological characteristics in Non-recurrent vs Recurrent HCC cohorts.

**Supplementary Table 2.** The molecular formula and CAS number of the single fatty acid methyl ester standard.

**Supplementary Table 3.** The antibodies used in IHC/IF.

**Supplementary Table 4.** Primers used for quantitative real-time PCR.

**Supplementary Table 5.** Clinical features and pathological findings in HCC patients profiled by snRNA-Seq (ANHC-3T and APHC-1T).

**Supplementary Table 6.** Peripheral blood lymphocyte subsets after surgery in the non-recurrent and recurrent groups.

**Supplementary Table 7.** Tacrolimus trough concentrations in recurrent versus non-recurrent groups.

**Supplementary Table 1.** Comparative Demographic, Clinical, and Tumor Pathological Characteristics in Non-recurrent vs Recurrent HCC Cohorts.

| **Characteristics** | **Non-recurrent group (n=23)** | **Recurrent Group (n=5)** | P-value |
| --- | --- | --- | --- |
| Gender |  |  |  |
| Male | 21 (91.3%) | 4 (80%) | 0.459 |
| Female | 2 (8.7%) | 1 (20%) |  |
| Age (years) | 50.00 (45.00-58.00) | 47.00 (38.50-61.00) | 0.613 |
| BMI (kg/m²) | 23.66 (19.59-26.56) | 21.24 (18.49-23.01) | 0.215 |
| ALT (U/L) | 30.00 (17.00-54.00) | 43.00 (17.50-77.50) | 0.805 |
| AST (U/L) | 42.00 (26.00-52.00) | 62.00 (35.50-96.00) | 0.168 |
| ALB (g/L) | 36.10 (32.60-39.60) | 35.20 (31.60-37.15) | 0.375 |
| TBIL (μmol/L) | 24.90 (12.50-65.80) | 28.00 (14.80-57.35) | 0.999 |
| γ-GT (U/L) | 45.00 (29.00-85.00) | 28.00 (18.50-117.5) | 0.652 |
| ln[AFP (ng/mL)] | 1.929 (1.075-3.833) | 4.511 (1.062-6.979) | 0.290 |
| Tumor size (cm) | 3.200 (2.500-5.800) | 6.700 (4.150-9.500) | 0.022* |
| HBV-DNA (IU/mL) |  |  | 0.051 |
| ≤ 50 | 20 (86.96%) | 2 (40%) |  |
| > 50 | 3 (13.04%) | 3 (60%) |  |
| Child-Pugh Score |  |  | 0.515 |
| A | 11 (47.83%) | 3 (60%) |  |
| B | 7 (30.43%) | 2 (40%) |  |
| C | 5 (21.74%) | 0 (0%) |  |
| TNM stage |  |  | 0.574 |
| I-II | 18 (78.26%) | 3 (60%) |  |
| III | 5 (21.74%) | 2 (40%) |  |
| Bridging locoregional therapy, n (%) |  |  |  |
| TACE | 15 (65.22%) | 4 (80%) | 0.999 |
| RFA | 5 (21.74%) | 0 (0%) | 0.549 |
| ALB: Serum Albumin; TBIL: Total Bilirubin; γ-GT: Gamma-Glutamyl Transferase; HBV-DNA: Hepatitis B Virus DNA; AFP: Alpha-Fetoprotein;TACE：Transarterial Chemoembolization; RFA：Radiofrequency Ablation. * P < 0.05 | | | |

**Supplementary Table 2.** The molecular formula and CAS number of the

single fatty acid methyl ester standard.

| **Fatty acid abbreviation** | **molecular formula** | **CAS number** |
| --- | --- | --- |
| C4:0 | C_5_H_10_O_2_ | 623-42-7 |
| C6:0 | C_7_H_14_O_2_ | 106-70-7 |
| C8:0 | C_9_H_18_O_2_ | 111-11-5 |
| C10:0 | C_11_H_22_O_2_ | 110-42-9 |
| C11:0 | C_12_H_24_O_2_ | 1731-86-8 |
| C12:0 | C_13_H_26_O_2_ | 111-82-0 |
| C13:0 | C_14_H_28_O_2_ | 1731-88-0 |
| C14:0 | C_15_H_30_O_2_ | 124-10-7 |
| C14:1 | C_15_H_28_O_2_ | 56219-06-8 |
| C15:0 | C_16_H_32_O_2_ | 7132-64-1 |
| C15:1 | C_16_H_30_O_2_ | 90176-52-6 |
| C16:0 | C_17_H_34_O_2_ | 112-39-0 |
| C16:1 | C_17_H_32_O_2_ | 1120-25-8 |
| C17:0 | C_18_H_36_O_2_ | 1731-92-6 |
| C17:1 | C_18_H_34_O_2_ | 75190-82-8 |
| C18:0 | C_19_H_38_O_2_ | 112-61-8 |
| C18:1n9t | C_19_H_36_O_2_ | 1937-62-8 |
| C18:1n9c | C_19_H_36_O_2_ | 112-62-9 |
| C18:2n6t | C_19_H_34_O_2_ | 2566-97-4 |
| C18:2n6c | C_19_H_34_O_2_ | 112-63-0 |
| C20:0 | C_21_H_42_O_2_ | 1120-28-1 |
| C18:3n6 | C_19_H_32_O_2_ | 16326-32-2 |
| C20:1 | C_21_H_40_O_2_ | 2390-09-2 |
| C18:3n3 | C_19_H_32_O_2_ | 301-00-8 |
| C21:0 | C_22_H_44_O_2_ | 6064-90-0 |
| C20:2 | C_21_H_38_O_2_ | 61012-46-2 |
| C22:0 | C_23_H_46_O_2_ | 929-77-1 |
| C20:3n6 | C_21_H_36_O_2_ | 21061-10-9 |
| C22:1n9 | C_23_H_44_O_2_ | 1120-34-9 |
| C20:3n3 | C_21_H_36_O_2_ | 55682-88-7 |
| C20:4n6 | C_21_H_34_O_2_ | 2566-89-4 |
| C23:0 | C_24_H_48_O_2_ | 2433-97-8 |
| C22:2 | C_23_H_42_O_2_ | 61012-47-3 |
| C24:0 | C_25_H_50_O_2_ | 2442-49-1 |
| C20:5n3 | C_21_H_32_O_2_ | 2734-47-6 |
| C24:1 | C_25_H_48_O_2_ | 2733-88-2 |
| C22:6n3 | C_23_H_34_O_2_ | 2566-90-7 |

**Supplementary Table 3.** The antibodies used in IHC/IF.

| **Antibody** | **Catalog Number** | **Company** | **Dilution** |
| --- | --- | --- | --- |
| Anti-human EHHADH | 26570-1 | Proteintech | 1:400 |
| Anti-human ACSL3 | HA601213 | HUABIO | 1:500 |
| Anti-human CD8 | ET1606-31 | HUABIO | 1:200 |
| Anti-human TNF -α | ab6671 | abcam | 1:200 |
| Anti-human CD4 | ab133616 | abcam | 1:200 |
| Anti-human FOXP3 | 98377S | Cell Signaling Technology | 1:100 |

**Supplementary Table 4.** Primers used for quantitative real-time PCR.

| **Gene** | **Primer Sequences** | **Forward (5′→3′)** |
| --- | --- | --- |
| TBP | Forward (5′→3′) | TGTATCCACAGTGAATCTTGGTTG |
|  | Reverse (5′→3′) | GGTTCGTGGCTCTCTTATCCTC |
| EHHADH | Forward (5′→3′) | CGGAGCATCGTGGAAAACAGCA |
|  | Reverse (5′→3′) | CCGAGTCTACAGCAATCACAGG |
| ACSL3 | Forward (5′→3′) | CTTTCTCACGGATGCCGCATTG |
|  | Reverse (5′→3′) | CTGCTGCCATCAGTGTTGGTTTC |
| FADS2 | Forward (5′→3′) | TGCAACGTGGAGCAGTCCTTCT |
|  | Reverse (5′→3′) | GGCACATAGAGACTTCACCAGC |
| IL-1β | Forward (5′→3′) | AGCTCGCCAGTGAAATGATGG |
|  | Reverse (5′→3′) | GTCCTGGAAGGAGCACTTCAT |
| IL-6 | Forward (5′→3′) | AGACAGCCACTCACCTCTTCAG |
|  | Reverse (5′→3′) | TTCTGCCAGTGCCTCTTTGCTG |
| IL-10 | Forward (5′→3′) | TCTCCGAGATGCCTTCAGCAGA |
|  | Reverse (5′→3′) | TCAGACAAGGCTTGGCAACCCA |
| SPP1 | Forward (5′→3′) | CGAGGTGATAGTGTGGTTTATGG |
|  | Reverse (5′→3′) | GCACCATTCAACTCCTCGCTTTC |
| PVR | Forward (5′→3′) | CCAGTGAGCACTCAGGTACA |
|  | Reverse (5′→3′) | GGGGTCTTCATCCATTGGGG |
| NECTIN2 | Forward (5′→3′) | GCTATGACTGGAGCACGACC |
|  | Reverse (5′→3′) | CAGCAGTAGCAATGATGGCG |
| VSIR | Forward (5′→3′) | AGGCAAAGATGCACCATCCA |
|  | Reverse (5′→3′) | TGCAGCCGTGATGTTTTCAC |
| CTLA4 | Forward (5′→3′) | CATGATGGGGAATGAGTTGACC |
|  | Reverse (5′→3′) | TCAGTCCTTGGATAGTGAGGTTC |
| TIGIT | Forward (5′→3′) | TGGTCGCGTTGACTAGAAAGA |
|  | Reverse (5′→3′) | GGGCTCCATTCCTCCTGTC |

**Supplementary Table 5.** Clinical features and pathological findings in HCC patients profiled by snRNA-Seq (ANHC-3T and APHC-1T).

| **Variables** | **ANHC-3T** | **APHC-1T** |
| --- | --- | --- |
| Age(y) | 58 | 65 |
| Sex | Male | Male |
| Performance Status | 0 | 0 |
| Child-Pugh | C | A |
| HBsAg | Positive | Positive |
| ALT(U/L) | ≤50 | ≤50 |
| Tumor Markers |  |  |
| AFP(ng/mL) | 11.79 | 91 |
| AFP-L3(ng/mL) | < 0.6 | 33.62 |
| PIVKA-II(ng/mL) | 195.2 | 3.14 |
| AFP-L3 Ratio | < 5 | 36.94 |
| TNM | Ⅱ | ⅢA |
| T | T2 | T3 |
| N | N0 | N0 |
| M | M0 | M0 |
| Macrovascular tumor thrombus | NO | NO |
| Edmondson-Steiner | Ⅲ | Ⅲ |
| IHC |  |  |
| GPC-3 | Positive | Positive |
| HSP70 | Positive | Positive |
| KI67 | 40 | 60 |
| Arg-1 | Positive | Positive |
| Heppar | Positive | Positive |
| Abbreviation: PS, Performance Status; ALT, alanine aminotransferase; AFP, alpha‐fetoprotein; AFP-L3, Alpha-Fetoprotein Lens culinaris agglutinin-reactive 3; PIVKA-II, Protein Induced by Vitamin K Absence or Antagonist-II ; IHC, Immunohistochemistry. | | |

**Supplementary Table 6.** Peripheral blood lymphocyte subsets after surgery in the non-recurrent and recurrent groups.

| **Lymphocyte subset** | **Frequency (%)** | | P - value | **Absolute count (cells/µL)** | | P - value |
| --- | --- | --- | --- | --- | --- | --- |
|  | **Non-recurrent group** | **Recurrent group** |  | **Non-recurrent group** | **Recurrent group** |  |
| LYMPH | 9.195  (3.318-12.74) | 9.295  (5.175-14.92) | 0.553 | 538.5  (374.3-1089) | 870.5  (804.0-1023) | 0.428 |
| T cells | 74.65  (66.66-83.47) | 63.02  (49.47-81.03) | 0.290 | 390.0  (278.5-827.3) | 638.5 (524.5-712.0) | 0.616 |
| CD4+ T | 44.45  (33.94-51.55) | 42.45  (27.63-54.79) | 0.891 | 231.0  (173.8-441.8) | 370.5 (276.0-444.8) | 0.211 |
| CD8+ T | 27.02  (20.36-33.69) | 19.59  (15.38-38.36) | 0.437 | 117.5  (91.50-312.5) | 177.5 (143.8-323.0) | 0.290 |
| B cells | 13.01  (4.263-17.52) | 12.30  (5.925-18.27) | 0.950 | 76.00  (31.75-182.3) | 124.5  (45.75-163.5) | 0.667 |
| NK cells | 6.835  (5.730-10.09) | 3.955  (2.308-27.59) | 0.248 | 62.00  (27.75-94.50) | 31.00  (18.25-311.5) | 0.553 |

**Supplementary Table 7.** Tacrolimus trough concentrations in recurrent versus non-recurrent groups.

| **Time Point** | **Non-recurrent group** | **Recurrent group** | ***P* - value** |
| --- | --- | --- | --- |
| Days 0–7 | 5.250 (3.700-11.13) | 5.133 (4.450-20.60) | 0.816 |
| Days 8–14 | 6.050 (5.133-8.700) | 6.500 (4.975-9.250) | 0.809 |
| Days 15–21 | 7.400 (5.400-8.667) | 6.300 (5.233-9.017) | 0.999 |
| Days 22–28 | 7.133 (6.033-9.233) | 7.400 (4.500-9.650) | 0.783 |
| Months 2–3 | 5.300 (2.600-7.567) | 2.733 (2.067-4.500) | 0.193 |
| Months 4–6 | 1.767 (1.133-2.967) | 1.467 (0.7667-1.717) | 0.096 |
| Months 7–12 | 1.700 (1.267-2.767) | 1.500 (0.850-1.633) | 0.178 |
| Months 13–24 | 1.467 (1.100-1.733) | 0.9667 (0.800-1.500) | 0.206 |
